# Comparison of Kirschner Wire Versus Screw Fixation in 1,2 Inter-Compartment Supra Retinaculum Artery Pedicle-Vascularized Bone Graft for Scaphoid Fractures

**DOI:** 10.7759/cureus.17533

**Published:** 2021-08-29

**Authors:** Asma Muhammad Ali, Muhammad Adil, Mirza Shehab A Beg, Osama T Ahmed

**Affiliations:** 1 Plastic and Reconstructive Surgery, Liaquat National Hospital and Medical College, Karachi, PAK

**Keywords:** scaphoid non-union, vascularized bone graft, 1-2 icsra dorsal radius bone graft, k wire fixation, screw fixation

## Abstract

Introduction: The purpose of the study was to compare the outcomes of the Kirschner wires (k-wires) versus screw fixation in 1,2 inter-compartment supra retinaculum artery (ICSRA) pedicle-vascularized bone graft for scaphoid non-union treatment.

Method: A retrospective study that included all patients with scaphoid nonunion underwent pedicle-vascularized bone graft and fixated with either k-wire or screw from 2010 through 2019 in the Department of Plastic and Reconstructive Surgery at Liaquat National Hospital and Medical College. Clinical outcomes were compared between k-wire and screw fixation, which were measured in terms of union rate, time of healing, pre and post-operative Disabilities of the Arm, Shoulder, and Hand (DASH) scores, range of motion at wrist, and grip strength of hand.

Results: A total of 33 patients were included in the study. All of them had scaphoid non-union and were treated with 1,2 ICSRA pedicle-vascularized bone graft; 20 patients had a fixation with k-wire and 13 with a screw. Radiological healing was achieved in 18 patients with k-wire and 11 patients with screw fixation, with healing rates of 90% and 84.6%, respectively. There was a significant decrease in DASH score postoperatively in both groups. Although no significant difference between the outcomes of both groups whether on union rate, DASH score, or range of motion at the wrist.

Conclusion: Hence, there is no significant difference in outcome between k-wire and screw fixation methods. We concluded k-wire as a more acceptable option with vascularized bone graft fixation as less technically demanding and low cost as compared to screw fixation.

## Introduction

Scaphoid nonunion occurs frequently in young adults as a result of unrecognized injury or failed initial treatment [1]. Incidence of nonunion is greater in the proximal fracture due to compromised vascularity and small size of fragment making its fixation difficult [2]. Bone graft and rigid fixation are the mainstay treatment of scaphoid nonunion. In recent studies, vascularized bone graft has shown a reduction in healing time and played a significant role in avascular necrosis or proximal pole fractures [3]. Hence, a vascularized bone graft is considered as primary treatment in proximal pole fracture with established scaphoid nonunion with union rate ranges between 27% and 100% [4-7].

Based on the Zeidemberg technique [8], 1,2 inter-compartment supra retinaculum artery (1,2 ICSRA) pedicle-vascularized bone graft has shown promising results in scaphoid nonunion [9]. Rigid fixation of vascularized graft in scaphoid nonunion is a key step in healing and functional outcomes. Straw et al. reported the worst result with a 27% union rate with vascularized bone graft but it was found that fixation of vascularized bone graft was done by single Kirschner wires (k-wire); single k-wire would have been considered the culprit as it may not withstand rotation. In some studies, rigid fixation of 1,2 ICSRA vascularized bone graft with screw has superior results as compared to k-wire [4,6]. Recent studies have shown no significant difference between k-wire compared to screw fixation. They place multiple k-wire in a convergent [7].

At our center, we have been performing 1,2 ICSRA pedicle-vascularized bone grafts for the treatment of scaphoid nonunion for more than a decade. Per-operatively, it always had taken more time in screw fixation and was difficult to fix small fragments of the proximal pole of the scaphoid. It was hypothesized that a relatively larger screw size as compared to a smaller vascularized graft may sabotage the vascularity of graft and structural damage. Generally, surgeons perform k-wire fixation in 1,2 ICSRA pedicle-vascularized bone grafts after failed screw fixation attempts as a fallback option. The lower cost, easy placement, and fallback option had led the surgeon to perform k-wire fixation in most of our cases. However, controversy remains regarding its stability, safety, and outcomes. Acknowledging less evidence of comparative literature between k-wire and screw fixation outcomes in 1,2 ICSRA pedicle-vascularized bone graft, we felt the need for a present study to compare functional outcomes and rate of healing in two groups of fixation. So we could perform evidence-based practice at our institute.

## Materials and methods

This was a retrospective study, conducted at Liaquat National Hospital. Ethical committee approval was taken from the institutional board before the commencement of data collection. Data were extracted from the hospital OPD registry. The hospital OPD registry contained manual records of all visiting patients and their follow-up records. Data were extracted between the year January 2010 to January 2019 to access all cases of scaphoid fracture treated with 1,2 ICSRA pedicle-vascularized graft fixated with either k-wire or screw.

Inclusion criteria comprised of age ranges 16-60 years, all scaphoid nonunion treated with pedicle-vascularized graft, and a minimum of six months follow-up postoperatively. The exclusion was based on surgical procedures other than 1,2 ICSRA pedicle-vascularized bone graft, any other associated scaphoid injury, any previous scaphoid surgery, failure to follow up in the last hospital out-patient department, and the presence of scaphoid nonunion advanced collapse. Indications for surgery were delayed presentation of scaphoid fracture, failure of previous conservative treatment, consistent pain with decreased range of motion, all proximal pole fracture, and radiological nonunion of scaphoid fracture.

Totally, 35 patients fell into the inclusion criteria. All patients were called and requested to visit the outpatient department for the last follow-up; 33 patients visited the outpatient department and were included in the study. All 33 patients were examined on the last visit for functional outcomes.

Range of motion at wrist joint was measured as the angle at radial deviation, ulnar deviation, wrist flexion, and ulnar flexion at both hands for comparison range of motion was measured by a two-arm goniometer [10].

The grip strength of the affected hand was measured and compared with the contralateral hand by using a sphygmomanometer, the grip strength was measured while the patient was sitting comfortably and arm was kept in shoulder adducted, elbow flexed at 90°, forearm in the neutral position, and wrist with 0º to 30º extension. The sphygmomanometer was calibrated and pre-inflated at 20 mmHg and the patient was asked to press the cuff to his/her maximum grip strength and pressure gauge reading was noted and compared with the contralateral hand.

Pre and post-op Disabilities of the Arm, Shoulder, and Hand (DASH) scores were calculated on the last visit as an outpatient and recorded [11]. Any tenderness and pain were noted. All healed scaphoids were pain-free on the last O-P-D visit. Biodata, hand dominance, radiological healing assessed by X-ray finding (loss of fracture lines), types of fracture, occupation, gender, time of radiologic healing, and nonunion (radiological nonhealing six months of surgery) was extracted from hospital follow-up records.

Being a retrospective study, we confronted certain limitations including unavailability of pre and post-op computed tomography, information regarding the presence of avascular necrosis of proximal pole of scaphoid at time of surgery, size of bone grafts used in surgery, time duration of surgery in either group of fixation, and recall of pre-op DASH scores by patients from past on the last follow-up.

Surgical technique

All patients had the same surgical procedure that was 1,2 ICSRA pedicle-vascularized bone graft which was fixed either with k-wire or screws; originally procedure was based on the Zaidemberg procedure [8].

All patients were operated on under general anesthesia with loupe magnification and tourniquet. A single senior surgeon performed all surgeries. A lazy S-shaped incision that was centered over the styloid process of radius allows exposure of scaphoid dorsoradially, preserves cephalic and superficial radial nerve while dissecting. 1,2 ICSRA was visualized generally on the first and second extensor compartment retinaculum; extensor retinaculum was incised while preserving 1,2 ICSRA. Corticocancellous bone graft from radial bone was marked along with pedicle. Pedicle dissection was carried out from the distal to the proximal radial artery continued to the graft site with an elevation of a perivascular cuff of soft tissue. The scaphoid capsule was opened under wrist extensors, the fracture was visualized, and the scaphoid bone was prepared for graft placement. Radial bone graft was harvested with a fine osteotome. Tourniquet was released to see the viability of graft and punctate bleeding of the scaphoid, placement of the vascularized graft, and fixation with k-wire under image guidance were done.

**Figure 1:**
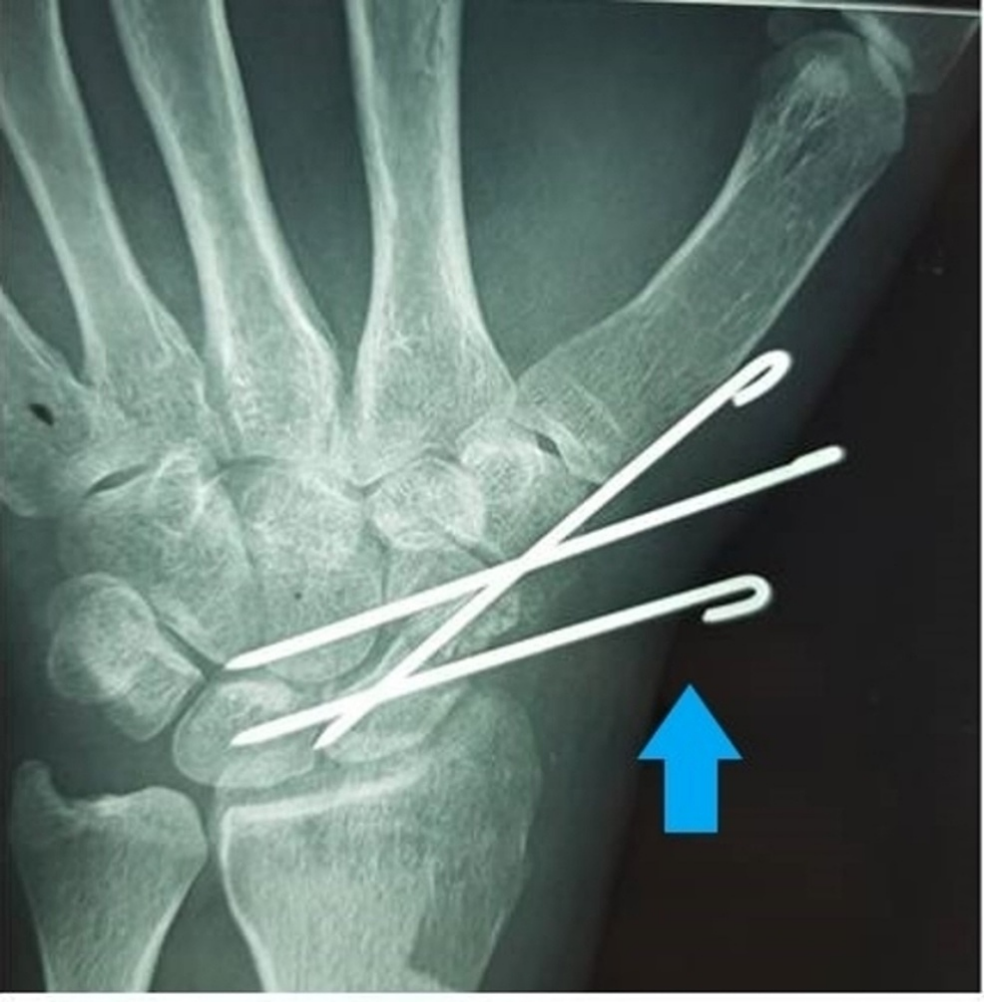
K-wire placement Two k-wires are already in convergence.

The number of k-wire placements (two or three) depends on stability achieved in the convergent manner assessed under image guidance [12], as shown in Figure 1. Internal screw fixation was done with a manual handheld driver. The back-slab was applied below the elbow up to six weeks post-operative to achieve complete immobilization.

Postoperatively patients were followed. Back-slab removed by six weeks. Postop rehabilitation started with active and passive range of motion of fingers with the continuation of wrist brace and thumb spica up to radiological healing k-wires were removed once signs of healing were achieved on X-rays. Once radiological healing was achieved on X-ray, wrist range of motion was started.

Statistical analysis

Patient data were compiled and analyzed through the Statistical Package for Social Sciences (SPSS) Version 25 (IBM Corp., Armonk, NY). Frequency and percentage were computed for qualitative variables. Means were calculated for the quantitative variable. The mean comparison was done by dependent and independent t-test. Association was checked by using the chi-square test and Fisher exact test as appropriate. P≤0.05 will be considered as significant.

## Results

This study included 33 patients. All patients had 1,2 ICSRA pedicle-vascularized bone graft for scaphoid nonunion; 20 patients were fixated with k-wire and 13 with screw with an average time to operation since injury was 8.2 months. All patients had preoperative pain and the mean DASH score of 77.2 ranges between 72 and 80.

There was no significant difference concerning age, gender, type of fracture, occupation, and time since injury had been found between k-wire and screw groups (Table 1).

**Table 1:** Demographic data comparison between k-wire and screw groups *Chi-square test was applied. **Fisher exact test was applied. ***Significant at 0.005.

Items		Screw group (n=13)	K-wire group (n=20)	Total (n=33)	P-value
Gender**	Male, n (%)	12(92.3)	18(90)	30(90.9)	1.000
	Female, n (%)	1(7.7)	2(10)	3(9.1)	
Occupation**	Machine operator, n (%)	1(7.7)	7(35)	8(24.2)	0.029***
	Driver, n (%)	2(15.4)	0(0)	2(6.1)	
	Factory worker, n (%)	1(7.7)	0(0)	1(3)	
	Farmer, n (%)	0(0)	1(5)	1(3)	
	Housewife, n (%)	0(0)	2(10)	2(6.1)	
	Labor, n (%)	3(23.1)	4(20)	7(21.2)	
	Student, n (%)	0(0)	4(20)	4(12.1)	
	Teacher, n (%)	3(23.1)	1(5)	4(12.1)	
	Delivery rider, n (%)	1(7.7)	0(0)	1(3)	
	A worker at a store, n (%)	2(15.4)	1(5)	3(9.1)	
Hand dominance**	Right, n (%)	12(92.3)	16(80)	28(84.8)	0.625
	Left, n (%)	1(7.7)	4(20)	5(15.2)	
Affected hand*	Right, n (%)	4(30.8)	10(50)	14(42.4)	0.275
	Left, n (%)	9(69.2)	10(50)	19(57.6)	
Fracture pattern**	Proximal, n (%)	7(53.8)	17(85)	24(72.7)	0.107
	Waist, n (%)	6(46.2)	3(15)	9(27.3)	
Union achieved**	Yes, n (%)	11(84.6)	18(90)	29(87.9)	1.000
	No, n (%)	2(15.4)	2(10)	4(12.1)	

The mean time from injury to surgery was eight months in both groups. The overall union rate was 87.7%. The rate of union achieved in k-wires compared to Herbert screw groups was insignificant with 90% and 84.6% in k-wire and screw groups, respectively (p-value=1.0) with a mean radiological healing time was 10 weeks in both groups. All patients achieved radiological healing and were pain-free. No statistical differences in postoperative clinical outcomes were found in both groups. Pre and post-operatively, there was a significant decrease in DASH scores in both groups (Table 2). Post-op range of motion and grip strength was comparable in both groups (Table 3).

**Table 2:** Significance decrease in DASH score post-op in both groups DASH: disabilities of the arm, shoulder, and hand.

	Pre-op DASH	Post-op DASH
K-wire	77.3	25.9
Screw	77.1	26.3

**Table 3:** Functional outcomes in both groups Independent t-test. SD: standard deviation.

	K-wire (%) mean±SD	Screw (%) mean±SD	p-Value
Ulnar deviation	85.13±4.19	83.40±6.38	0.353
Radial deviation	79.37±5.02	78.97±6.18	0.842
Wrist extension	88.74±3.53	88.73±3.43	0.994
Wrist flexion	91.51±3.29	91.09±2.38	0.691
Grip strength	90.00±7.43	92.69±6.95	0.306

Four out of 33 patients did not achieve radiological healing and remained painful on clinical examination. Here also, no significant differences were seen in both groups. Humpback deformity was found in two nonunion. Not all patients were followed for the long term, minimum follow-up was six months. Non-healed scaphoids were pain-free and had joint arthritic changes. Two non-healed scaphoids were collapsed.

## Discussion

Scaphoid non-union treatment has been always difficult to manage due to various factors affecting its healing [13]. The mainstay treatment of scaphoid nonunion remained bone graft and fixation (immobilization). Vascularized over non-vascularized bone grafts are used for early consolidation but still, ambiguous situations persist that a bone graft is superior to another. Munk and Larson [7] showed the superiority of vascular bone graft with 91% union rates with vascularized bone graft as compared to 84% with non-vascularized bone graft. Other studies also report higher union rates with vascularized bone graft 100% [5] and 86% [3]. On the basis of superior union rates with vascularized graft, we at our center preferred vascularized bone graft for scaphoid nonunion. The present study has shown union rates of 87.7% with 1,2 ICSRA pedicle-vascularized bone grafts.

However, Straw et al. reported the worst result of a 27% union rate with vascularized bone graft. Might be single k-wire would not have achieved desired immobilization in cases reported by Straw et al. [4].

Fixation of vascularized bone graft can be done by Herbert screw, AO mini screw, and k-wire [1]. Recent fixation techniques for vascularized bone graft have also been introduced, which is a combination of k-wire and external fixator [14].

In this study, we had performed screw and k-wire fixation in pedicle-vascularized bone grafts. Screws have significant resistance properties for bending as compared to k-wire but could not withstand multiaxis and cyclical rotation as k-wire does [15]. That is why our protocol remained to keep immobilized until radiological healing is achieved in either type of fixation, though this affects a post-op range of motion of the wrist and return to work but our rehabilitation protocol helps to achieve desirable effects. The prime advantage of the Herbert screw is a non-protrusive head [16]. Although good surgical expertise is needed for the right placement, malposition may occur which may lead to osteoarthritic changes in the future [17]. An unsuccessful attempt may damage the articular surface of the proximal pole. Technical difficulty and size of proximal pole fragment make Herbert screw less suitable for proximal pole fracture nonunions. K-wire is a simpler technique, which ensures rotational stability and can also be used for internal fixation for a small fragment of the scaphoid. Although K-wire does not provide lag compression to fracture, the thickness of the k-wire should be taken into account as there is a risk of breakage and should be removed before starting mobilization.

In the present study, the number of k-wire fixations was 20 as compared to screw fixation which was 13. This unequal distribution was due to vascularized bone graft fixation outcomes that were studied retrospectively. Selection of fixation method whether k-wire or screw was depended on availability, affordability, and technical challenging case at the time of surgery.

It has been shown that there is a longer union time with k-wire fixation and less predictability than Herbert screw. In a meta-analysis, they reported union rate with k-wire as compared to Herbert screw 77% and 94%, respectively, with an average union time of 20 weeks [18]. In contrast in the present series, statistical analysis did not show a significant difference in k-wire and screw fixation with significantly early healing, both group's average healing time is 10 weeks. Hence, this proved achievement of significant stability with the placement of multiple k-wires in a convergent manner works effectively. This allows covering more surface area of the scaphoid with less damage to a small fragment of the proximal pole. Similarly, we have found other studies also showed no significant differences in union rate with k-wire and screw with mean union time of 14 weeks [10]. Meisel et al. [19] showed 100% union rates with a similar technique of k-wire fixation in scaphoid nonunion with iliac bone graft. Though the slightly higher number of union with k-wire as compared to Herbert screw in the present study are consistent with Munk et al. [7] results, they also reported in a systemic review of 147 publications, where k-wire fixation estimated a higher union rate as compared to screw fixation that is 91% versus 88%, respectively. This higher union rate was also persisted in vascularized graft fixation of the scaphoid with 94% and 87% for k-wire and screw respectively. Our study also showed a slightly higher percentage of union with k-wire as compared to screw fixation 90% and 87%, respectively. In our understanding, a higher rate with k-wire is a consequence of less damage to small fragments of a proximal pole and vascularized bone grafts which are already vulnerable due to compromised blood supply [20,21].

To achieve union is not the only criterion for assessing the result of treatment of scaphoid-nonunion. Reduction in pain, better range of motion, and prevention of subsequent progress to osteoarthritis along with union are the goal of scaphoid nonunion treatment [22]. In our study, we compared the functional outcome of both groups in terms of pre and post-op DASH scores in both groups; there is a significant decrease in DASH scores as shown in Table 2. Other parameters were a range of motion at the wrist joint and grip strength which were compared with the contralateral hand. There was no significant difference in both groups wrist extension, flexion, and grip strength was achieved up to 90% of the contralateral hand. Although, radial deviation was only achieved by less than 80% of the contralateral hand (Table 3). These outcomes are comparable with another study, in which vascularized fixation was mainly done by screw [3]. No significant difference in outcomes of both techniques of fixation has made us believe that k-wire is a better option as less expensive, less technical expertise required, and more suitable for smaller fracture or vascularized bone graft fragments. K-wire is simple and less expensive and is a good alternative to Herbert screw in developing countries.

Our study had some limitations. As a retrospective study, single-center, we could not assess scaphoid heights postoperatively.

## Conclusions

Delayed diagnosis of scaphoid fracture and retrospective nature of its blood supply has rendered it vulnerable to scaphoid nonunion. Despite we performed two different fixation techniques, immobilization and fixation remain the major component of its treatment. The present study demonstrates that the Kirschner wire fixation option is safe and reliable, with nearly comparable bony union rates and functional outcomes. No difference is seen in union rate, time of radiological healing, and DASH scores in either type of fixation. Compression screw fixation is technically more demanding than k-wires. Being part of the developing world, the cost has always influenced treatment and management. Concerns and worries of many surgeons to use K-wires may get a new perspective with this study. Easy placement and less expensive k-wires are seemed to be a good alternative to compression screws in developing countries. Further prospective studies are needed in this field for a definite clarification of outcome and results between k-wire and screw fixation in 1,2 ICSRA pedicle-vascularized bone graft.
